# Functional Dysregulations in CA1 Hippocampal Networks of a 3-Hit Mouse Model of Schizophrenia

**DOI:** 10.3390/ijms22052644

**Published:** 2021-03-05

**Authors:** Solenn Percelay, Jean-Marie Billard, Thomas Freret, Annie Andrieux, Michel Boulouard, Valentine Bouet

**Affiliations:** 1 UNICAEN, INSERM, COMETE, CYCERON, CHU Caen, Normandie Université, 14000 Caen, France; jean-marie.billard@inserm.fr (J.-M.B.); thomas.freret@unicaen.fr (T.F.); michel.boulouard@unicaen.fr (M.B.); valentine.bouet@unicaen.fr (V.B.); 2 Inserm U1216, CEA, Grenoble Institut Neurosciences, Université Grenoble Alpes, 38000 Grenoble, France; annie.andrieux@univ-grenoble-alpes.fr

**Keywords:** psychiatric disorder, hippocampus, functional plasticity, long term potentiation, NMDA receptor

## Abstract

For a better translation from treatment designs of schizophrenia to clinical efficiency, there is a crucial need to refine preclinical animal models. In order to consider the multifactorial nature of the disorder, a new mouse model associating three factors (genetic susceptibility—partial deletion of the *MAP6* gene, early-life stress—maternal separation, and pharmacological treatment—chronic Δ-9-tetrahydrocannabinol during adolescence) has recently been described. While this model depicts a schizophrenia-like phenotype, the neurobiological correlates remain unknown. Synaptic transmission and functional plasticity of the CA1 hippocampal region of male and female 3-hit mice were therefore investigated using electrophysiological recordings on the hippocampus slice. While basal excitatory transmission remained unaffected, NMDA receptor (NMDAr)-mediated long-term potentiation (LTP) triggered by theta-burst (TBS) but not by high-frequency (HFS) stimulation was impaired in 3-hit mice. Isolated NMDAr activation was not affected or even increased in female 3-hit mice, revealing a sexual dimorphism. Considering that the regulation of LTP is more prone to inhibitory tone if triggered by TBS than by HFS, the weaker potentiation in 3-hit mice suggests a deficiency of intrinsic GABA regulatory mechanisms. Indeed, NMDAr activation was increased by GABA_A_ receptor blockade in wild-type but not in 3-hit mice. This electrophysiological study highlights dysregulations of functional properties and plasticity in hippocampal networks of 3-hit mice, one of the mechanisms suspected to contribute to the pathophysiology of schizophrenia. It also shows differences between males and females, supporting the sexual dimorphism observed in the disorder. Combined with the previously reported study, the present data reinforce the face validity of the 3-hit model that will help to consider new therapeutic strategies for psychosis.

## 1. Introduction

Schizophrenia is a debilitating pathology that affects approximately 1% of the population. It is characterized by symptoms described as positive and negative, accompanied by cognitive deficits [1]. While treatments with typical and atypical antipsychotics are relevant for positive symptoms, they are questioned for negative symptoms and poorly effective for cognitive impairments [2,3]. One major obstacle for the development of potent new treatments against schizophrenia is the absence of relevant and fully recognized animal models of the disorder. Indeed, even if a wide range of rodent models already exists, their construct validity may be questioned since they are mostly based on one sole risk factor [4,5,6]. Using a sole factor does not fit with the idea that schizophrenia would result from a combination of genetic vulnerability and neurodevelopmental insults affecting neurotransmission systems [7]. It has been therefore recommended to use animal models with the highest construct validity respecting this multifactorial origin [8,9,10,11]. In this context, a 3-hit mouse model has recently been described, which associates a genetic susceptibility with an early developmental stress and a late pharmacological insult [12]. More precisely, a partial deletion of the microtubule-associated protein *MAP6* gene, a transient maternal separation (for 24 h at the age of 9-days), and a chronic administration of Δ-9-tetrahydrocannabinol (THC, 8 mg/kg daily during adolescence) have been associated [13,14,15], postulating that the three factors will combine and synergize to trigger the mimicking-disorder. A recent investigation indicated that this 3-hit model displays some key-elements of the negative- and cognitive-like deficits expected: sociability deficit and cognitive deficits such as working memory impairment and alteration of reference memory, and a decrease in hippocampus volume [12]. Knowing whether changes in functional properties of synapses occur in 3-hit mice represents a major milestone to progress our knowledge of the pathophysiology of schizophrenia. In addition, investigating males and females in order to take into account the sexual dimorphism reported in patients, which is rarely done in animal models, is of importance [16,17].

The present study reveals alterations of both glutamate and GABA-mediated regulation of functional plasticity at CA3/CA1 hippocampal synapses of 3-hit mice that are differentially expressed in males and females. Together with the recently provided characterization, our functional analysis argues to consider the 3-hit conditioning as a relevant strategy to investigate schizophrenia-related phenotype.

## 2. Results

### 2.1. Basal Synaptic Transmission

**Figure 1:**
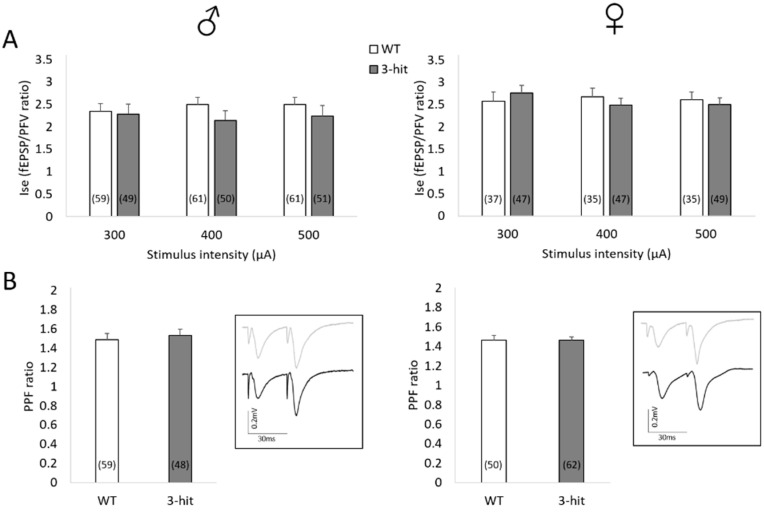
Basal synaptic transmission in CA1 hippocampal area is not impaired in 3-hit mice. (**A**) Index of synaptic efficacy Ise (fEPSP/PFV ratio) in Wild Type (WT) and 3-hit mice, males (left) and females (right), with increasing stimulus intensity. (**B**) Paired pulse facilitation (PPF) ratio in WT and 3-hit mice, males (left) and females (right). In inserts, traces recorded are shown: WT (grey line) and 3-hit (black line) male (left) and a female (right) mouse. The number of slices used in each group is indicated in histogram bars. Represented data are Mean ± SEM.

The synaptic efficacy of basal non-N-methyl-D-aspartate receptor (non-NMDAr)-mediated neurotransmission was considered in a first step. This was assessed by determining field excitatory postsynaptic potentials/presynaptic fiber volleys ratio (fEPSP/PFV ratio) thus considered as an index of synaptic efficacy (Ise). Ise calculated for three increasing stimulus intensities was not statistically different in 3-hit mice compared to Wild Type (WT) mice, neither in males nor in females (Figure 1A). Additionally, no difference between males and females was found when WT or 3-hit groups were considered, respectively.

### 2.2. Paired-Pulse Facilitation

Basal neurotransmission closely depends on presynaptic release of glutamate, which was assessed by the paired-pulse facilitation (PPF) sowed paradigm (see Figure 1B, insert). As illustrated in Figure 1B, PPF ratio was not statistically different between WT and 3-hit mice. Furthermore, no difference in PPF ratio between males and females was shown within each experimental group.

Taken together, these results indicate that the 3-hit conditioning does not impact the basal neuronal communication in CA1 hippocampal networks, neither in males nor in females.

### 2.3. Functional Plasticity

In a second step, the effects of 3-hit conditioning on the expression of functional plasticity in CA1 hippocampal networks have been considered using different paradigms of tetanus stimulation.

**Figure 2:**
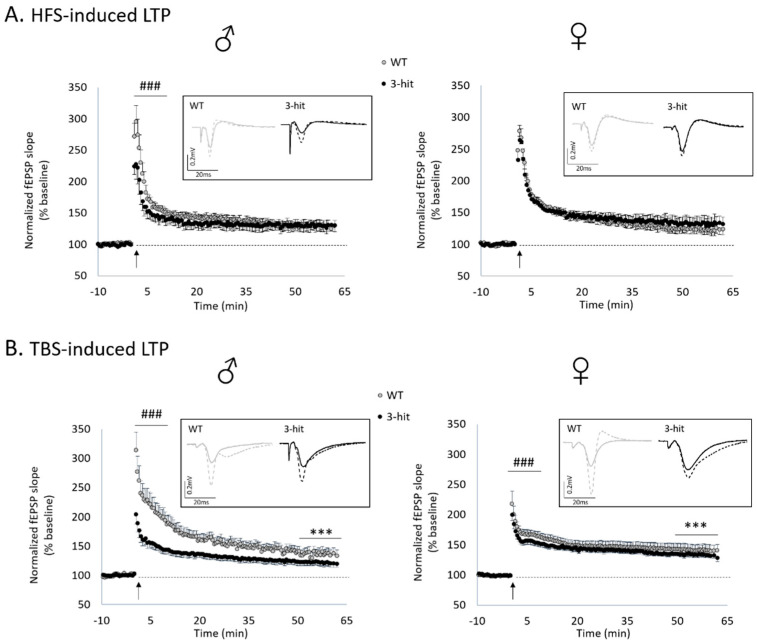
Functional plasticity is altered in 3-hit mice according to stimulation protocols, sex and/or the timing of potentiation. (**A**) Time-course of high frequency stimulation (HFS)-induced long-term potentiation (LTP) in WT and 3-hit mice, males (left) (WT: n = 13 slices vs. 3-hit group n = 11 slices) and females (right) (WT: n = 13 slices vs. 3-hit: n = 12 slices). (**B**) Time-course of theta-burst stimulation (TBS)-induced LTP in WT and 3-hit mice, males (left) (WT: n = 26 slices vs. 3-hit group n = 13 slices) and females (right) (WT: n = 35 slices vs. 3-hit: n = 21 slices). In inserts, examples of traces recorded in a WT and a 3-hit mouse before (filled line) and 60 min after (dashed line) the conditioning stimulation. Represented data are Mean ± SEM; non-parametric permutation test (group and time effect), group effect ***: *p* < 0.001 (last 15 min). ANOVA with permutation tests for STP, group effect ###: *p* < 0.001 (first 10 min).

High frequency stimulation (HFS)-related conditioning stimulation induced long-term potentiation (LTP) in WT and 3-hit mice of both genders (Figure 2A) since the differences between the baseline and the last 15 min of recordings were significant in all experimental groups (*p* < 0.001). The magnitude of HFS-induced LTP was not different between WT and 3-hit male (WT: 129.4 ± 8.0% of baseline vs. 3-hit: 130.2 ± 9.6%, mean ± SEM; neither group effect, time effect or time x group interaction) and female mice (WT: 123.9 ± 6.7% of baseline vs. 3-hit: 132.7 ± 10.9%, mean ± SEM; neither group effect, time effect or time x group interaction) (Figure 2A). Furthermore, comparison of male vs. female WTs showed higher amplitude in the former (*p* < 0.001). No difference was found between male and female 3-hit.

Theta-burst stimulation (TBS) also induced a long-lasting potentiation of synaptic transmission of both WT and 3-hit groups (Figure 2B). Indeed, statistical analysis between the baseline and the last 15 min of recordings showed a significant increase in fEPSP magnitude in all experimental groups (*p* < 0.001). However, in contrast to HFS, when intergroup comparison was considered, the magnitude of TBS-induced LTP was significantly lower in the 3-hit group than in the WT. This decrease was found in both males (WT: 138.8 ± 8.9% of baseline vs. 3-hit: 122.8 ± 6.2%, mean ± SEM; group effect *p* < 0.001, neither time effect or time x group interaction) and females (WT: 141.5 ± 9.6% of baseline vs. 3-hit: 134.0 ± 4.6%, mean ± SEM; group effect *p* < 0.001, neither time effect or time x group interaction) (Figure 2B). Furthermore, by comparing male and female, we found higher amplitude in females in the WT group as well as in the 3-hit group (*p* < 0.001).

Statistical differences for the two paradigms of conditioning stimulations were also found for the first 10 min post tetanus. After HFS, a significant decrease in this short-term potentiation (STP) occurred in 3-hit males (*p* < 0.001) but not in 3-hit females compared to WTs (Figure 2A). Whereas STP magnitude was similar in male and female WT mice, females 3-hit have higher STP than males (*p* < 0.001).

On the contrary, STP was significantly decreased in both male and female 3-hit mice compared to WT after TBS (*p* < 0.001), as observed for LTP expression (Figure 2B). In this stimulation protocol, no difference in STP magnitude was found between males and females in the WT group as well as in the 3-hit group.

### 2.4. NMDAr Activation

Considering the pivotal role of NMDAr in the induction and expression of short- and long-term functional plasticity at synapses of neuronal networks, we looked for changes in NMDAr activation in males and females of the different experimental groups. This was achieved by comparing fEPSPs isolated in low Mg^2+^ medium supplemented with the non-NMDAr antagonist NBQX (10 μM).

**Figure 3:**
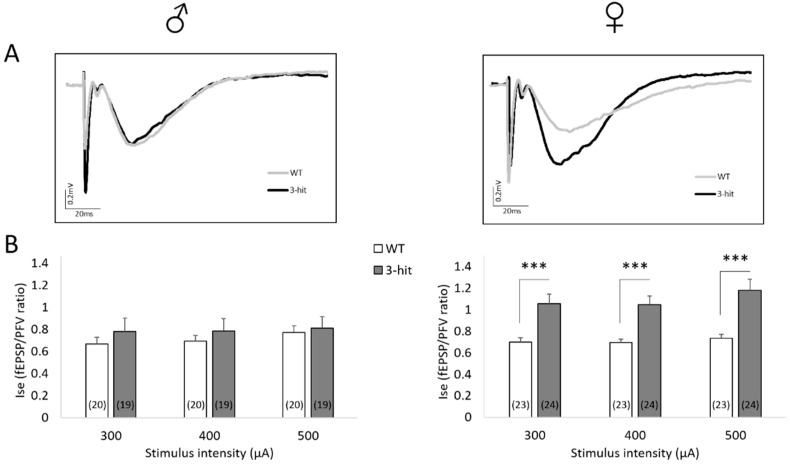
(**A**) NMDAr activation is specifically enhanced in female 3-hit mice. Superimposed traces of long lasting NMDAr-induced fEPSPs recorded in a slice from a male (left) and a female (right), WT (grey line) and a 3-hit (black line) mouse. (**B**) NMDAr-mediated synaptic efficacy ratio Ise (fEPSP/PFV ratio) in WT and 3-hit male (left) and female (right) mice with increasing stimulus intensity. Number of slices per group are indicated in histogram bars. Represented data are Mean ± SEM; ANOVA or ANOVA with permutation tests, ***: *p* < 0.001.

In these conditions, long-lasting fEPSPs specifically mediated by NMDAr were recorded (Figure 3A). Interestingly, a sexual dimorphism was observed since the Ise was not impacted in 3-hit males compared to WT regardless of the intensity of stimulation, whereas it was significantly higher in females (*p* < 0.001), indicating a specific gender-related enhancement of NMDAr activation (Figure 3B). No difference between male and female WTs was shown. However, as displayed in Figure 3, females 3-hit have higher NMDAr-mediated synaptic efficacy ratio than males 3-hit (*p* < 0.001).

**Figure 4:**
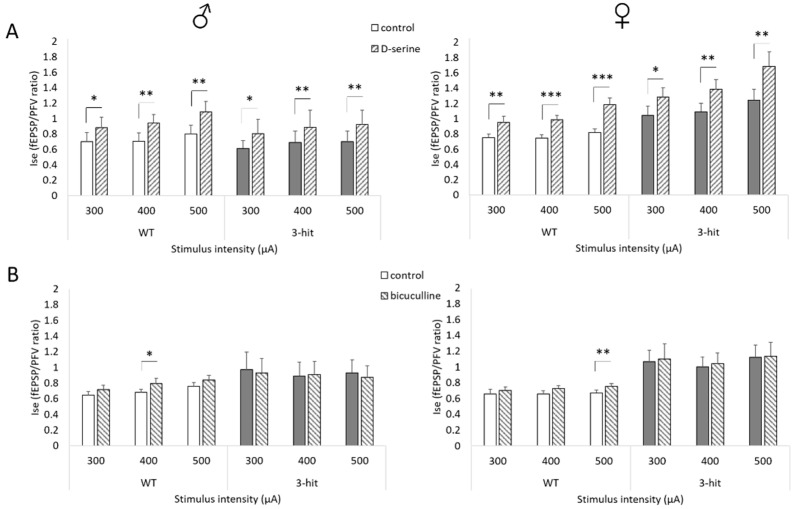
Responsiveness of NMDAr activation to bicuculline but not D-serine is altered in 3-hit mice. (**A**) Index of NMDAr-mediated synaptic efficacy Ise (fEPSP/PFV ratio) determined in slices from WT and 3-hit male (left) and female (right) mice, with increasing stimulus intensity, in control vs. D-serine supplemented artificial cerebrospinal fluid (aCSF) (males: WT: n = 18 slices vs. 3-hit: n = 20 slices; females: WT: n = 12 slices vs. 3-hit: n = 10 slices). (**B**) Index of NMDAr-mediated synaptic efficacy Ise (fEPSP/PFV ratio) determined in slices from WT and 3-hit male (left) and female (right) mice, with increasing stimulus intensity, in control vs. bicuculline supplemented aCSF (males: WT: n = 11 slices vs. 3-hit: n = 9 slices; females: WT: n = 13 slices vs. 3-hit: n = 12 slices). Represented data are Mean ± SEM; Paired t-tests or Wilcoxon paired t tests, *: *p* < 0.05; **: p < 0.01; ***: *p* < 0.001.

In the CA1 hippocampal area, NMDAr activation requires the binding of the co-agonist D-serine in addition to glutamate [18]. Since the basal excitatory neurotransmission was not affected in 3-hit mice, one may consider that synaptic availability of glutamate was not impacted in those animals. An altered occupancy of the NMDAr co-agonist binding site by D-serine could then induce changes in NMDAr activation. To test this possibility, a saturating concentration of D-serine (100µM) was added to the artificial cerebrospinal fluid (aCSF) [19]. Consequently, a significant increase in Ise was observed in all experimental groups regardless of the intensity of stimulation (*p* < 0.05) (Figure 4A). The percentage increase by exogenous D-serine was similar in male and female WT and 3-hit mice (Figure S1A), indicating that the occupancy level of the NMDAr glycine binding sites was not impacted by the 3-hit conditioning. Furthermore, it is interesting to note that in this condition of maximal recruitment of NMDAr, no statistical differences in the Ise were found between WT and 3-hit male mice whereas it remained significantly higher in 3-hit females (Figure S2) (*p* < 0.05), suggesting that the synaptic NMDAr density could be altered in the 3-hit model depending on the gender.

We found that HFS- and TBS-induced LTP were differentially impacted in male and female 3-hit mice. Considering that the regulation by GABA inhibitory tone is different for the expression of these two forms of long-lasting synaptic potentiation [20], we looked for changes in inhibition by comparing the effects of pharmacological GABA_A_ receptor blockade on NMDAr activation. Adding the GABA_A_ receptor antagonist bicuculline (10 µM) to the low Mg^2+^ and NBQX-supplemented aCSF increased the Ise in both male and female WT groups, regardless of the intensity of stimulation (Figure 4B and Figure S1B). On the contrary, the Ise was not impacted by bicuculline administration in both male and female 3-hit mice (Figure 4B and Figure S1B), suggesting a decrease in the inhibitory tone in the animal model.

## 3. Discussion

This electrophysiological study investigated the 3-hit model of schizophrenia-like phenotype that has been recently described [12] associating three major risk factors including a genetic susceptibility, an early stressful event, and a late pharmacological insult. We report selective functional deficits (dysregulation of NMDAr- and GABA-related processes) in male and female 3-hit mice that recapitulate several neurotransmission system alterations that have been related to the schizophrenia-like phenotype, thus reinforcing the face validity of the animal model [7,21,22].

The 3-hit model was based on a combination of genetic, environmental, and pharmacological risk factors. Each of these factors is not known to be sufficient per se to reproduce all aspects of the disorder, but are supposed to generate a more extensive pathology if they are associated [23]. Although a lot of studies showed a substantial role of genetic susceptibility on schizophrenia prevalence [24,25], whether one particular gene locus in the patients’ genome is essential remains unknown. In the 3-hit model, a partial deletion of the *MAP6* gene, which regulates the intracellular microtubule organization [13,26] was selected rather than a complete knock-out, considering that heterozygous *MAP6* mice present slight cognitive and social deficits [13,27,28]. Indeed, complete deletion of *MAP6* drives severe behavioral and functional disturbances mimicking symptoms of psychosis [29,30,31]. Additionally, environmental perturbations in childhood have been correlated to a stronger risk to develop a psychiatric disease [32,33]. An early maternal separation in rodents thus disturbs brain development and adult behavior [14,34,35]. Furthermore, a decrease in social interaction after maternal separation for 24 h was reported in mice [34]. Therefore, maternal separation at PND9 for 24 h was retained for the 3-hit model. Finally, THC administrations from PND32 to PND52 were used as a third factor in the 3-hit model since cannabis increases the risk of developing psychotic-like symptoms in case of chronic consumption during adolescence [36,37]. In rodents, deleterious effects of adolescent exposure with cannabinoid receptor 1 agonists have been reported in adult behavior (see review [38]), and THC administration affects long-term memory and increases anxiety states [39]. Furthermore, THC adolescent exposure is widely used in rodent schizophrenia models as a single factor [40] or in combination with another factor [17].

The present electrophysiological study first indicates that the 3-hit conditioning does not significantly affect the basal synaptic transmission mediated by non-NMDAr, i.e., AMPA subtype of glutamate receptors, in CA1 hippocampal networks. This is strengthened by the absence of changes in PPF, indicating that presynaptic release of glutamate is not altered.

Regarding functional plasticity, contradictory results exist in the literature. Transcranial magnetic stimulation showed a reduced LTP-like plasticity in the cortex of schizophrenic patients [41,42]. In rodent models of schizophrenia, some studies reported a weaker hippocampal LTP after social isolation [43], deletion of dysbidin-1 [44], or treatment with the NMDAr antagonist MK801 [45], whereas an increase in LTP has been reported after deletion of DISC1 [46]. What is more, no change was found in rats after prenatal injection of methylazoxymethanol acetate, a neurodevelopmental model of schizophrenia [47]. Finally, different consequences on functional plasticity depending on stimulation protocols have been reported [48], more particularly in rodent models of schizophrenia [49,50]. In the present study, we observed changes in the expression of functional plasticity in 3-hit mice depending not only on the strength of the conditioning stimulation but also on the gender of the animals and/or the type of plasticity (STP vs. LTP). Indeed, HFS-induced LTP was unchanged in 3-hit mice, whereas the expression of TBS-induced long-lasting potentiation was reduced regardless of the gender of the animals. This last result is consistent with Chalkiadaki et al. [51] investigating a developmental model of schizophrenia using methylazoxymethanol administration during gestation in mice. One striking difference between the HFS and TBS stimulation paradigms is the impact of GABA modulation that is critical for TBS but not HFS-induced LTP to be expressed [20]. In contrast to WT, we found that NMDAr activation in 3-hit mice was not affected by GABA receptor blockade with the GABA_A_ antagonist bicuculline, suggesting a failure of inhibitory influences in CA1 neuronal networks. In control conditions, presynaptic GABA_B_ autoreceptors are critically involved in the TBS-induced LTP, allowing a long-lasting potentiation to develop by substantially diminishing GABA release [20]. If GABA availability is compromised in 3-hit mice, we could speculate that a weaker activation of GABA_B_ autoreceptors could promote a potent inhibition to persist during the TBS delivery, thus reducing the expression of subsequent LTP. Specific changes in GABA_B_ receptor activation now need to be determined in 3-hit mice. However, since *MAP6* knock-out mice showed a decrease in vesicle density in presynaptic terminals in the CA1 area [29], alteration of presynaptic release during the TBS delivery cannot be definitively ruled out, although there were no changes in the PPF paradigm of glutamate release in 3-hit mice.

It is interesting to note that the impaired GABA regulation of hippocampal network functioning could be linked to an attenuated PPI response [52,53]. This key behavioral feature, that characterizes animal models of schizophrenia, has been recently described in 3-hit mice (see supplementary data of Bouet et al. (2020)) [12] and represents a behavioral correlation to the present functional deregulation, also seen in *MAP6* knock-out mice [54].

We found that HFS-induced LTP was not altered in 3-hit mice. In this paradigm of much stronger conditioning stimulation than the TBS, the contribution of GABA regulation is minimized and the number of NMDAr per se located at synaptic connections is the critical parameter that governs the magnitude of the potentiation. Interestingly, a greater maximal isolated NMDAr activation was found in 3-hit mice but only in females, possibly reflecting a higher synaptic receptor density. Gender-related differences are gaining more and more importance in preclinical studies [16,17], and some sexual dimorphisms have been shown in hippocampal plasticity [55,56,57,58]. Even if synaptic NMDAr activity increased in female 3-hit mice, this facilitation did not change the level of HFS-induced LTP. Furthermore, we showed that only male but not female 3-hit mice displayed a lower short-term potentiation in the HFS protocol. Whether STP and LTP are two independent forms of functional plasticity, and may be associated with distinct aspects of memory formation, remains an open issue [59]. However, their differential alterations could contribute, at least in part, to the sexual dimorphism associated with schizophrenia-like cognitive deficits. Indeed, whatever the direction in changes affecting the expression of functional plasticity in neuronal networks of 3-hit mice, it is interesting to note that they parallel with alterations of hippocampus-related behaviors. In fact, these mice display working and reference memory impairments, sociability deficits, and PPI impairments [12], that were currently associated with hippocampal structural alterations and dysfunctions [52,53,60,61,62,63,64].

In conclusion, we provide evidence of functional impairments of hippocampal networks (inhibitory dysregulation and impaired glutamate-related synaptic plasticity) in 3-hit mice displaying deficits generally associated with the pathophysiology of schizophrenia [41,42,43,44,45,65,66]. Multifactorial models such as the 3-hit model used here, certainly represent the future for preclinical studies of psychiatric disorders and therefore appear as the best strategy for the development of more relevant human medication, notably those targeting the GABAergic and glutamatergic systems.

## 4. Materials and Methods

Experiments were performed in accordance with French and European Economic Community guidelines for the care and use of laboratory animals (2010/63/UE, project n° 2019050617054190_v2, date of approval 2019/09/18). All efforts were made to minimize the number of animals used as well as their suffering. In the present study, a total of 42 mice were used, 14 males and 10 females Wild Type (WT), and 6 males and 12 females 3-hit.

### 4.1. Genetic Susceptibility

Heterozygous *MAP6* (MAP6+/−) and WT mice were obtained from breeding between male MAP6+/− mice (129svPas/C57BL/6 strain, from Centre Universitaire de Ressources Biologiques de l’Université de Caen Normandie (CURB), France) and female WT mice (C57BL/6 strain, from Janvier Labs, France). Animals were kept in groups of 3 to 5 individuals in a reversed 12 h/12 h light/dark cycle (light on at 19 p.m., off at 7 a.m.), with regulated temperature (21 ± 1 °C) and humidity (55 ± 10%). Food and water were given ad libitum. At weaning, mice were identified with a subcutaneous chip placed under anaesthesia (isoflurane (5–2.5%) in an O_2_/N_2_O mixture (0.3/0.7)), and tail sampling was performed for genotyping [13].

### 4.2. Maternal Separation (MS)

Half of the litters were separated from their mother for a single period of 24 h on the 9th post-natal day (PND9). The mothers were removed and individually placed near the pups in another cage from 9:00 a.m. to 9:00 a.m. the following day. Then, the mothers were returned to their litters and left undisturbed until weaning. Control mice were submitted to the same protocol but separated for only 20 s [14,34].

### 4.3. Δ-9-Tetrahydrocannabinol Treatment

Tetrahydrocannabinol (THC, Sigma Aldrich^®^, Lyon, France) was dissolved in a vehicle saline solution (0.9%) with polysorbate 80 (Tween 80, Sigma-Aldrich, Steinheim, Germany, 3.5%) to obtain a final dose of 8 mg/kg [15]. The 3-hit mice received daily intraperitoneal THC solution (at 5:00 pm) from PND32 to PND52 while WT mice received the vehicle (3.5% Tween 80 in saline solution).

### 4.4. Ex Vivo Electrophysiology

Between PND140 to PND256, mice were anesthetized with isoflurane and decapitated. The brain was rapidly removed from the skull and placed in chilled (−3 to 0 °C) artificial cerebrospinal fluid (aCSF) containing 124 mM NaCl, 3 mM KCl, 1.5 mM MgSO_4_, 2.5 mM CaCl_2_, 26.2 mM NaHCO_3_, 1.2 mM NaH_2_PO_4_, and 11 mM glucose. Transverse slices (400 μm thick) of both ventral and dorsal part of the hippocampus were obtained with a slicer (Mc Ilwain^®^) and placed in aCSF in a holding chamber, at 28 °C, for at least one hour before recordings [67]. A slice was then transferred to a submersion-type recording chamber and continuously perfused at room temperature with aCSF equilibrated with 95% O_2_, 5% CO_2_. Extracellular recordings were obtained from glass micropipettes (2–5 MΩ) filled with 2 M NaCl positioned in the stratum radiatum of the CA1 hippocampal area. PFVs and non-NMDA-mediated fEPSPs were evoked in control aCSF by electrical stimulation of the Schaffer collaterals/commissural pathway located in the stratum radiatum. The slope of three successive PFVs and fEPSPs was determined using WinLTP^®^ software [68,69,70]. To evaluate the level of synaptic activation, an index of synaptic efficacy (Ise) corresponding to the fEPSP/PFV ratio was calculated and plotted systematically against stimulus intensity (300, 400 and 500µA). Paired-pulse facilitation (PPF) of synaptic transmission was then induced to investigate possible changes in presynaptic mechanisms of glutamate release using paired pulse with inter-stimulus interval of 30 ms. PPF was determined as the ratio of the slope of the second response over that of the first fEPSP. In addition, specific NMDA receptor (NMDAr) activation was evaluated by recording fEPSPs pharmacologically isolated from slices perfused with aCSF containing low Mg^2+^ (0.1 mM) and supplemented with the non-NMDAr antagonist 2,3-dioxo-6-nitro-1,2,3,4-tetrahydrobenzoquinoxaline-7-sulfonamide (NBQX, 10µM). The effects of exogenous application of the NMDAr co-agonist D-serine (100µM) or the GABA_A_ receptor antagonist bicuculline (10µM) were assessed by comparing the fEPSP/PFV ratio before and 15 min after the addition of the drug to the aCSF.

In order to investigate long-term potentiation (LTP) and short-term potentiation (STP) of synaptic transmission, a test stimulus was applied every 10 s in control medium and adjusted to get a fEPSP with a baseline slope of 0.1 V/s. The averaged slope of 3 fEPSPs was measured for 15 min before the delivery of a high-frequency stimulation (HFS), consisting of 1 burst at 100 Hz pulses delivered for 1 s. In another set of experiments, a theta-burst stimulation (TBS) was delivered, consisting of 4 successive sequences delivered at 0.1 Hz. Each sequence consisted in 5 bursts separated from each other by 200 ms (5 Hz). Within each burst, 4 impulses were delivered at 100 Hz [48]. In both HFS and TBS-related experiments, testing with single pulse was then resumed for 60 min to determine the level of potentiation. STP was quantified by comparing the first 10 min after the stimulation to the baseline while LTP was quantified with the last 15 min of recording, e.g., between 45 and 60 min after the conditioning stimulation.

All pharmacological agents were diluted directly in the perfusion medium from stock solutions prepared in distilled water. All drugs were purchased from Tocris Bioscience^®^ (Bristol, UK).

### 4.5. Data Analysis

Statistical analyses were performed using R software 3.6.2 (Free Software Foundation, Vienna, Austria; R Development Core team 2009). Shapiro tests were used to assess data normality. When data were normally distributed, ANOVAs were used for group comparisons, and paired t-tests to assess drugs effects. When data were not normally distributed, ANOVAs with permutations were used for group comparison, and Wilcoxon paired tests to assess drugs effects. Concerning LTP and STP analyses, group comparisons were performed with non-parametric permutation test ezPerm function with time effect (package “ez”). Differences were considered significant when the associated p value was below 0.05.

## Data Availability

The data presented in this study are available on request from the corresponding author. The data are not publicly available because they belong to our funding.

## Supplementary Materials

The following are available online at https://www.mdpi.com/1422-0067/22/5/2644/s1, Figure S1: Increase in NMDAr activation by exogenous bicuculline but not D-serine is lower in 3-hit mice, Figure S2: Maximal synaptic NMDAr activation is higher in female 3-hit mice.
