# Supplementary material for: Functional Dysregulations in CA1 Hippocampal Networks of a 3-Hit Mouse Model of Schizophrenia
Source: Int J Mol Sci. 2021 Mar 5;22(5):2644. doi: 10.3390/ijms22052644 (PMC7961987; doi:10.3390/ijms22052644)
Supplement: Supplementary file 1 [file ijms-22-02644-s001.pdf]

Supplementary Materials: The following are available online at www.mdpi.com/xxx/s1, Figure S1: Increase in NMDAr activation by exogenous bicuculline but not D-serine is lower in 3-hit mice, Figure S2: Maximal synaptic NMDAr activation is higher in female 3-hit mice.

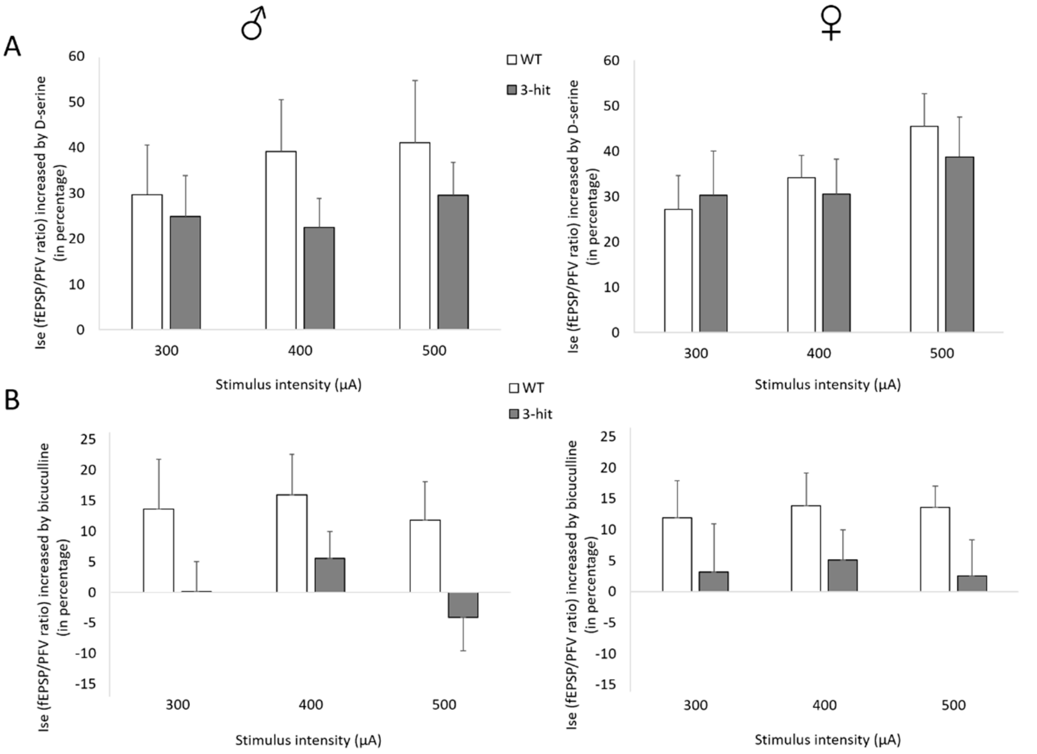

Figure S1. Increase in NMDAr activation by exogenous bicuculline but not D-serine is lower in 3-hit mice. (**A**) Percentage increase in the index of NMDA-r-mediated synaptic efficacy Ise (fEPSP/PFV ratio) in WT and 3-hit slices from male (left) and female (right) mice with increasing stimulus intensity in control vs. D-serine supplemented aCSF (males: WT: n = 18 slices vs. 3-hit: n = 20 slices; females: WT: n = 12 slices vs. 3-hit: n = 10 slices). (**B**) Percentage increase in the index of NMDAr-mediated synaptic efficacy Ise (fEPSP/PFV ratio) in WT and 3-hit slices from male (left) and female (right) mice with increasing stimulus intensity in control vs. bicuculline supplemented aCSF (males: WT: n = 11 slices vs. 3-hit: n = 9 slices; females: WT: n = 13 slices vs. 3-hit: n = 12 slices). Represented data are Mean ± SEM; ANOVA or ANOVA with permutation tests.

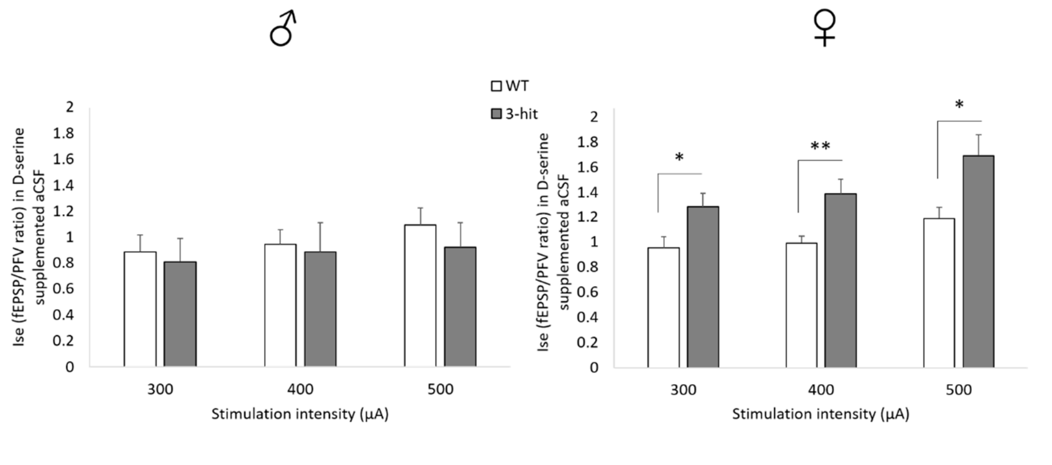

Figure S2. Maximal synaptic NMDAr activation is higher in female 3-hit mice. Index of NMDAr-mediated synaptic efficacy Ise (fEPSP/VA ratio) in hippocampal slices from WT and 3-hit from male (**left**) and female (**right**) mice with increasing stimulus intensity in D-serine supplemented aCSF (males: WT: n = 18 slices vs. 3-hit: n = 20 slices; females WT: n = 12 slices vs. 3-hit: n = 10 slices). Represented data are Mean ± SEM; ANOVA or ANOVA with permutation tests, *: $p < 0.05$; **: $p < 0.01$.
